# Divergent chondro/osteogenic transduction laws of fibrocartilage stem cell drive temporomandibular joint osteoarthritis in growing mice

**DOI:** 10.1038/s41368-023-00240-5

**Published:** 2023-08-25

**Authors:** Ruiye Bi, Qianli Li, Haohan Li, Peng Wang, Han Fang, Xianni Yang, Yiru Wang, Yi Hou, Binbin Ying, Songsong Zhu

**Affiliations:** 1 State Key Laboratory of Oral Diseases & National Center for Stomatology & National Clinical Research Center for Oral Diseases & Department of Orthognathic and TMJ Surgery, West China Hospital of Stomatology, Sichuan University, Chengdu, China; 2 State Key Laboratory of Oral Diseases & National Center for Stomatology & National Clinical Research Center for Oral Diseases & West China Hospital of Stomatology, Sichuan University, Chengdu, China; 3 Department of Stomatology, Ningbo First Hospital, Ningbo, China

**Keywords:** Cell biology, Stem-cell differentiation

## Abstract

The anterior disc displacement (ADD) leads to temporomandibular joint osteoarthritis (TMJOA) and mandibular growth retardation in adolescents. To investigate the potential functional role of fibrocartilage stem cells (FCSCs) during the process, a surgical ADD-TMJOA mouse model was established. From 1 week after model generation, ADD mice exhibited aggravated mandibular growth retardation with osteoarthritis (OA)-like joint cartilage degeneration, manifesting with impaired chondrogenic differentiation and loss of subchondral bone homeostasis. Lineage tracing using *Gli1-CreER*^*+*^*;* *Tm*^*fl/-*^mice and *Sox9-CreER*^*+*^*;Tm*^*fl/-*^mice showed that ADD interfered with the chondrogenic capacity of *Gli1*^+^ FCSCs as well as osteogenic differentiation of *Sox9*^+^ lineage, mainly in the middle zone of TMJ cartilage. Then, a surgically induced disc reposition (DR) mouse model was generated. The inhibited FCSCs capacity was significantly alleviated by DR treatment in ADD mice. And both the ADD mice and adolescent ADD patients had significantly relieved OA phenotype and improved condylar growth after DR treatment. In conclusion, ADD-TMJOA leads to impaired chondrogenic progenitor capacity and osteogenesis differentiation of FCSCs lineage, resulting in cartilage degeneration and loss of subchondral bone homeostasis, finally causing TMJ growth retardation. DR at an early stage could significantly alleviate cartilage degeneration and restore TMJ cartilage growth potential.

## Introduction

The complex function of the synovial joint is largely determined by the efficient coordination of different structures within the intraarticular capsule. In most synovial joints of the body, the fibrocartilage is located between the articulating bones, which provides functions of shock absorption, cushioning between bones, as well as smoothing joint movement.^1,2^ Under abnormal stress caused by structural abnormalities, such as anterior disc displacement (ADD) in the temporomandibular joint (TMJ), meniscus tear in the knee joint, and femoroacetabular impingement in the hip joint, the coordination of the joint structure starts to be broken, and osteoarthritis (OA) happens.^3–5^

OA is one of the most devastating chronic diseases that affects 7% of the global population, more than 500 million people worldwide.^6^ The increased prevalence of OA in the young population has drawn considerable attention as it affects the patient’s joint development during the growth period, which may lead to severe skeletal dysfunction and deformities. For instance, TMJOA in adolescents often interferes with mandibular condyle growth, progresses to loss of joint function, joint instability, and malocclusion, and results in dento-maxillofacial deformities.^7–11^

Numerous clinical investigations showed that the onset of TMJOA is related to TMJ ADD.^12–19^ The condyle-disc stability is seen as the key factor for smooth movement, which is essential for normal masticatory functions, including talking, chewing, swallowing, and facial expression. Therefore, once the disc is displaced, these pathological conditions of the disc often act as antecedents to a series of degenerative changes that can affect the entire TMJ.^7,8^ At the same time, a series of animal studies also identified that experimentally induced TMJ ADD using rabbits and rats could lead to OA-like cartilage degeneration and subchondral bone loss and cause mandibular growth retardation.^20–23^ However, these studies are limited to the endpoint change of chondrocyte and osteoblast/osteoclast lineage, the definite cellular and molecular origins of the OA process in TMJ ADD are still fogged.

Recently, studies have revealed that the fibrocartilage stem cells (FCSCs) population is resided in TMJ cartilage in different species,^24–27^ which cells were found with critical roles as cell resources for TMJ homeostasis and repair,^25,28^ and were able to interact with endothelial cells in vascularized bone formation.^26^ At the adult stage, the cell features of FCSCs were regulated by various signaling pathways such as Wnt, Notch, and TNF-α/Nf-κB.^25,29,30^ At the growth stage, besides the phenomenon that FCSCs can differentiate toward chondrocytes, though, how the cell fate of FCSCs is regulated by OA during TMJ cartilage growth remains to be elucidated.

In this current study, we chose to investigate the functional role of FCSCs in TMJOA during the growth period. To accomplish this purpose, we, for the first time, generated an ADD-induced TMJOA mouse model with FCSCs lineage specifically labeled. Thus we were able to mimic the joint degeneration phenotype at the growth period and elucidate spatiotemporal rules of FCSCs for cartilage growth under both physiological and pathological conditions. We further generated a TMJ disc reposition (DR) mouse model, investigating the potential clinical application of surgical DR for adolescent TMJOA patients.

## Results

### TMJ ADD in growing mice led to mandibular growth retardation with OA-like degeneration of TMJ cartilage

**Fig. 1:**
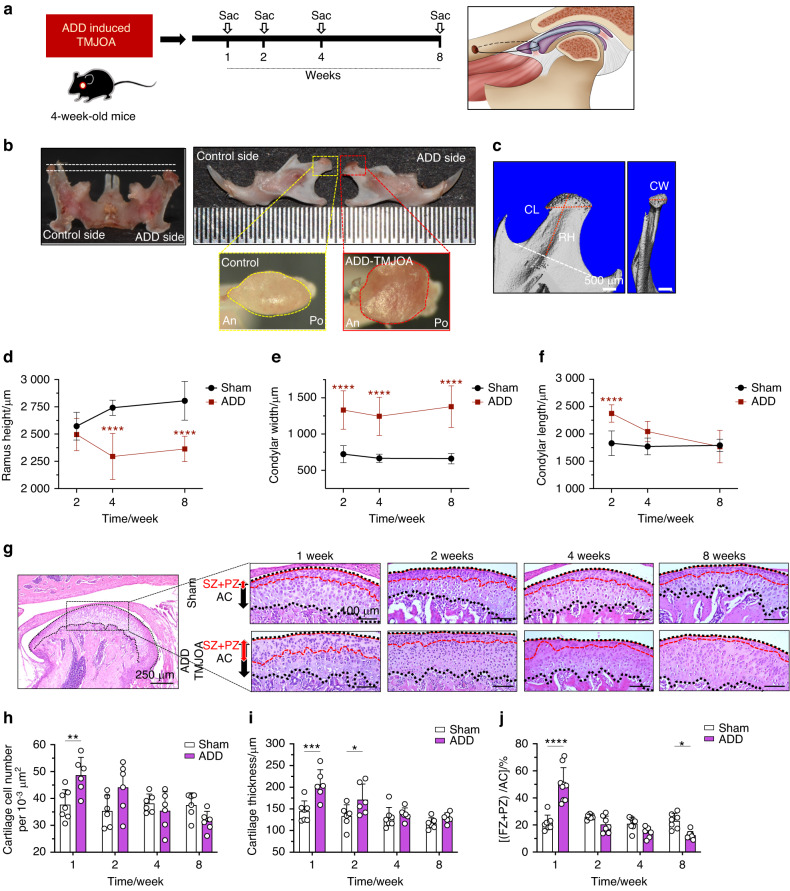
TMJ ADD in growing mice led to mandibular growth retardation. **a** Schematic diagram of ADD-induced TMJOA mouse model generation and timing of animal sacrifice after ADD surgery. **b** Representative images of morphological changes of ADD condyle compared to the control condyle. **c** To analyze the condyle growth of ADD mice, mandibles were dissected and scanned by microCT and were reconstructed at 2/4/8 weeks post-surgery. **d**–**f** The ramus height (RH), condylar width (CW), and condylar length (CL) of ADD mice were analyzed. *n* = 5–7. ****P < 0.0001. The horizontal line and error bar indicates mean ± SD. **g** H&E staining of TMJ condylar cartilage 1/2/4/8 weeks after ADD surgery. FZ fibrocartilage zone, PZ proliferative zone, AC articular cartilage. **h**–**j** The semi-quantification of cell number in articular cartilage, the thickness of articular cartilage, and the ratio of (FZ + PZ) thickness/articular cartilage thickness. *N* = 6–9. *P < 0.05, **P < 0.01, ***P < 0.001, ****P < 0.000 1

The successful establishment of ADD mouse model was identified by observing that sutures in front of the zygomatic arch were kept intact, no fractures of the zygomatic arch occurred, and the posterior band of the disc was located in front of the apex of the condylar head (Fig. S1a). The mandibular midline shifted to the non-operative side immediately after model generation and was aligned with the upper teeth midline in a week (Fig. S1b). The ADD mice were also found with a slower body weight increase in the first 2 weeks after model generation, possibly due to occlusion change and joint pain. Two weeks later, the body weight of ADD mice became comparable to Sham mice during the observation period (Fig. S1c). The anteriorly displaced disc was found with perforation in 37.5% of the mice at 1 week, in 40% of the mice at 2 weeks, and in 100% of the mice at 4 weeks and 8 weeks after model generation (Table 1). From 2 weeks to 8 weeks, the TMJ growth on ADD side was found retarded, manifesting as aggravated ramus height (RH) loss compared to Sham TMJ at 4 weeks and 8 weeks (Fig. 1a–d). However, the increased condylar width (CW) of ADD side was found during the entire observational period. The condylar length (CL) was markedly increased at 2 weeks and was gradually decreased at later stages (Fig. 1e, f). These phenotypes signified a pathological bone remodeling of the condylar head in growth retarded mouse ADD TMJ.

**Table 1:** The perforation ratio of ADD mice at different stages

Weeks after model generation	Perforated disc number (*n*)/ total disc number (*n*)
1	3/8
2	4/6
4	6/6
8	6/6

To further investigate the histological changes of mouse ADD TMJ, hematoxylin and eosin (H&E) staining and Safranin O staining were performed. Compared to the Sham condylar cartilage with a clear hierarchy of cell layers, the ADD cartilage at 1 week showed a significantly disarranged cell distribution with increased cell numbers and layer thickness, especially in the superficial layer (fibrocartilage zone (FZ) + proliferative zone (PZ)) (Fig. 1g–j). From 2 weeks to 8 weeks, the cartilage surface of ADD TMJ became rough, and the disarrangement of cell distribution was aggravated (Fig. 1g).

**Fig. 2:**
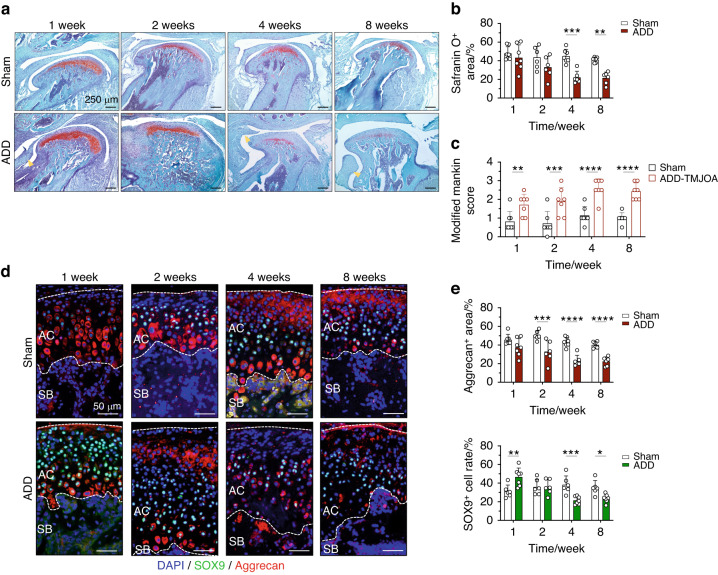
TMJ ADD caused OA-like degeneration in cartilage in growing mice. **a** Safranin O staining of TMJ cartilage at 1/2/4/8 weeks after ADD surgery. Yellow arrows indicated the anterior displaced TMJ disc. **b** Semi-quantification of Safranin O^+^ area in ADD mice at different stages. *N* = 6–7. **c** Modified Mankin score of mice TMJ cartilage after ADD-induced TMJOA model generation at different stages. *N* = 7–8. **d** Immunofluorescent staining of Aggrecan and SOX9 expressions in the articular cartilage of ADD-TMJOA mouse at 1/2/4/8 weeks after ADD surgery. AC articular cartilage, SB subchondral bone. **e** Semi-quantitative of Aggrecan^+^ area and SOX9^+^ cell numbers in the articular cartilage. *P < 0.05, **P < 0.01, ***P < 0.001, ****P< 0.000 1

In the Sham mice, the safranin O^+^ matrix was found uniformly distributed in the deep zone of the cartilage. In ADD mice with the disc released and pulled forward from the top of the condyle (Fig. 2a, yellow arrow), the safranin O^+^ matrix distribution became disordered. At 1 week, there were both abundant safranin O^+^ chondrocytes and increased safranin O secretion in the matrix of the posterior region of the condyle in ADD mice, while both the number of chondrocytes and expression of safranin O were dramatically decreased from 2 weeks till later stages (Fig. 2a, b). In addition, the Modified Mankin scores of the ADD joints gradually increased after model generation. These phenotypes revealed that the ADD surgery induced an OA-like cartilage degradation phenotype in TMJ cartilage (Fig. 2c).

To further investigate the cell fate transformation and functional changes of chondrocytes under ADD-TMJOA, immunofluorescent co-staining of Aggrecan and SOX9 was performed in ADD mice at different stages. In the ADD-TMJOA group, the Aggrecan expression was mainly found assembled in the middle region and posterior region of the condylar cartilage. At 1 week, the Aggrecan^+^ matrix area was not statistically different between ADD group and the Sham group. However, SOX9 expression in ADD group was remarkably up-regulated (Fig. 2d, e), which signifies increased chondrogenic transduction of cartilage progenitors toward chondrocytes to maintain normal matrix secretion under OA conditions. At later stages (from 2 weeks-8 weeks), both Aggrecan^+^ matrix area and SOX9^+^ cell rates were significantly decreased in ADD-TMJOA mice (Fig. 2d, e). These findings demonstrate that the compensatory effect of increased chondrogenic differentiation was lost at later stages of ADD-TMJOA, which resulted in the destruction cartilage matrix, interrupted chondrogenic differentiation, and OA progress.

### ADD-TMJOA led to subchondral bone loss and remodeling in condylar growth

**Fig. 3:**
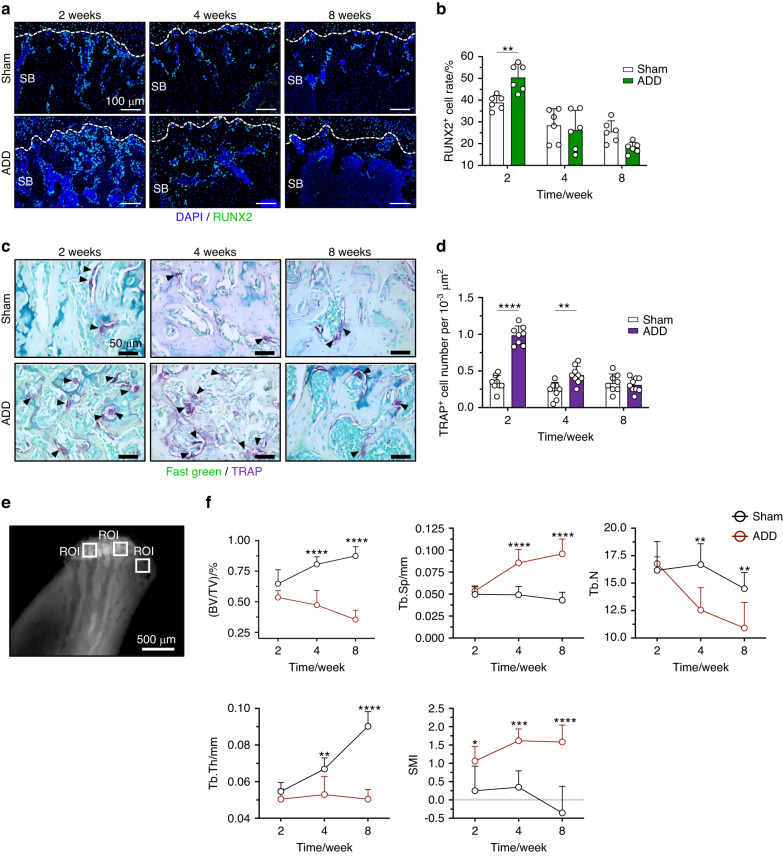
TMJ ADD caused subchondral bone loss in growing mice. **a** Immunofluorescent staining of RUNX2 expressions in the subchondral bone area of TMJ in ADD-TMJOA mice at 2/4/8 weeks after ADD surgery. SB subchondral bone. **b** Semi-quantification of RUNX2^+^ cell ratio in TMJ SB area. **c** TRAP staining in the TMJ subchondral bone area. The black arrow indicated TRAP^+^ cells. **d** Semi-quantification of TRAP^+^ cell numbers in the SB area of ADD mice. *N* = 6–8. **e** Representative image of microCT analysis of ADD TMJ condyle. and selections for analysis. The white square indicates the region of interest (ROI). **f** The quantification analysis of ROI. BV/TV(%): bone volume fraction; Tb.Sp (mm): trabecular separation; Tb.N (1/mm): trabecular number; Tb.Th (mm): trabecular thickness; SMI structure model index. *N* = 6–7. *P < 0.05, **P < 0.01, ***P < 0.001, ****P < 0.000 1

The condylar cartilage is the center of mandibular growth and development. As ADD caused the initiation of TMJOA in growing mice, the subchondral bone formation would be affected as well. Therefore, we examined the changes in subchondral bone by immunofluorescent staining of RUNX2. In the Sham group, RUNX2^+^ cell numbers were found gradually decrease in the subchondral bone area during mice growth after ADD model generation. In the ADD-TMJOA group, the RUNX2^+^ cell number was significantly upregulated at 2 weeks and was rapidly reduced at 4 weeks and 8 weeks, which was not statistically different from that in the Sham group (Fig. 3a, b). Tartrate-resistant acid phosphatase (TRAP) staining showed that the numbers of osteoclasts (indicated by black arrows in Fig. 3c) were significantly increased in the ADD-TMJOA group at 2 weeks and were degressive at later stages. At 8 weeks, the osteoclast number of the ADD group was not significantly different from the Sham group (Fig. 3c, d). At the same time, microCT analyses showed that the structure model index (SMI) of ADD mice was increased at 2 weeks, which signified the trabecular micro-structure was changed and the bone loss initiated at this early stage. Then the bone volume over total volume (BV/TV), the trabecular thickness (Tb.Th), and the trabecular number (Tb.N) in ADD subchondral bone were gradually decreased at 4 weeks and 8 weeks, as well as increased trabecular separation/spacing (Tb.Sp) at the later stages (Fig. 3e-f). These results indicate that ADD-TMJOA leads to abnormally activated trabecular bone turnover and remodeling at the early stage and finally causes subchondral bone loss during the growth of TMJ condylar cartilage.

### ADD-TMJOA leads to the activation of the chondrogenic capacity of *Gli1*^+^ FCSCs

**Fig. 4:**
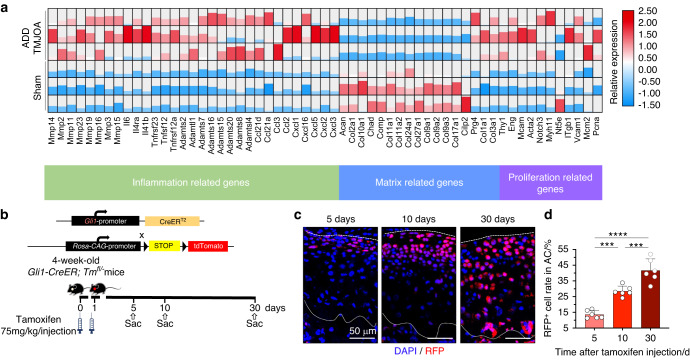
FCSCs proliferation was stimulated by ADD-TMJOA. **a** The heatmap of mRNA expressions of inflammation-related genes, cartilage matrix-related genes, and proliferation-related genes in mice condylar cartilage at 1 week after ADD surgery. **b** Mating tactics for generating *Gli1-CreER*^*+*^*; Tm*^*fl/-*^ mice. **c** Representative images of RFP^+^ cell distributions in TMJ cartilage at 5/10/30 days after tamoxifen injection. **d** Semi-quantification of RFP^+^ cell numbers in articular cartilage at 5/10/30 days after tamoxifen injection. *N* = 6. ***P < 0.001, ****P < 0.000 1

To clarify the critical signaling pathways that contribute to the pathological changes of cartilage and subchondral bone in ADD-TMJOA mice, we performed mRNA bulk sequencing using TMJ cartilage samples from ADD mice and Sham mice at 1 week. The bulk sequencing analysis showed a total of 3356 differential genes between the 2 groups, including 1398 up-regulated genes and 1958 down-regulated genes in the ADD-TMJOA samples. Notably, the mRNA expression of OA-related cytokines (*Tnfrsfs*, *Ccls*, etc.) was significantly increased under ADD-TMJOA (Fig. 4a). Consistently, the cartilage matrix-related genes (*Acan*, *Col2a1*, etc.) were also downregulated, indicating disequilibrium of cartilage matrix homeostasis (Fig. 4a). At the same time. The cell proliferation-related genes (*Thy1*, *Pcna*, etc.) were upregulated in ADD group, which phenotype hints us the function of chondrogenic progenitors were activated in ADD-TMJOA cartilage at the early stage (Fig. 4a).

Therefore, we are interested in the fate change of cartilage progenitors under ADD-TMJOA. We used *Gli1* as the specific marker of FCSCs, which was found as the main cartilage progenitor in TMJ.^27,31^ *Gli1-CreER*; *Rosa26*^*tdTomato*^ (*Gli1-CreER; Tm*^*fl/*^^−^) mice were used for FCSCs lineage tracing. 4-week-old *Gli1-CreER; Tm*^*fl/*^^−^ mice were injected with tamoxifen and were sacrificed at 5 days, 10 days, and 30 days (Fig. 4b). Five days after tamoxifen (TM) injection, red fluorescence^+^ (RFP^+^) lineage cells were found mainly distributing in the superficial layer, consist 10% of the cells in the articular cartilage (Fig. 4c, d). RFP^+^ lineage cells continued to proliferate and gradually migrated to the deep layers. Thirty days after TM injection, 36% of the cells in articular cartilage were RFP^+^, which was proved to be derived from the FCSCs lineage (Fig. 4c, d).

**Fig. 5:**
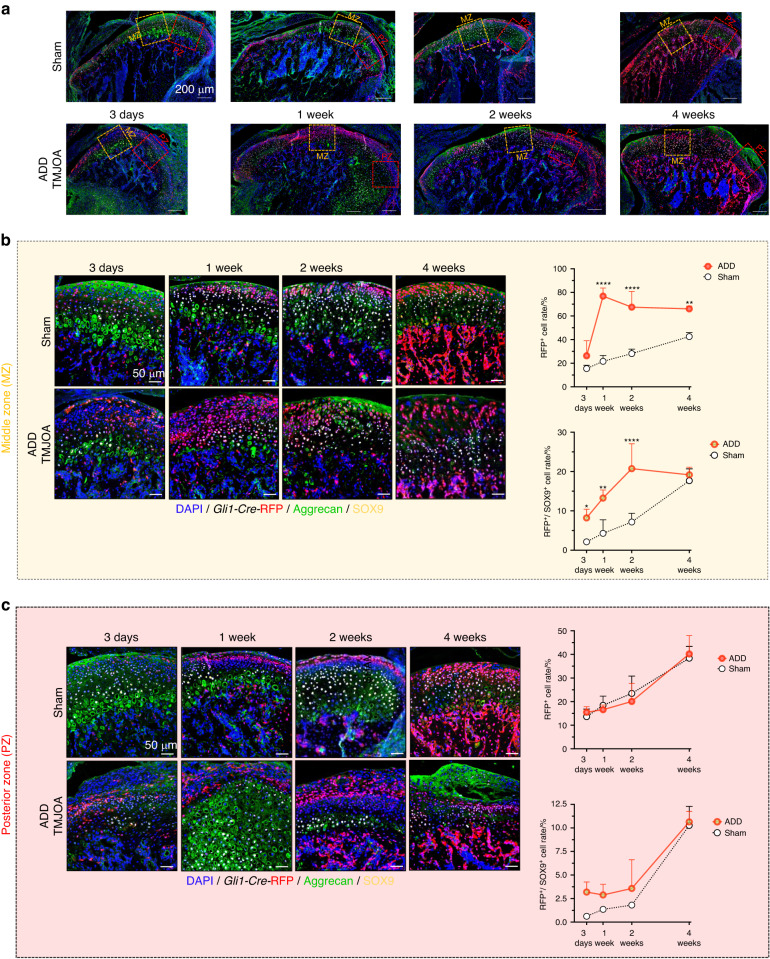
ADD-TMJOA led to activation of the chondrogenic capacity of *Gli1*^+^ FCSCs. **a** Expressions of *Gli1*-RFP, Aggrecan, and SOX9 were analyzed by immunofluorescent staining in TMJ cartilage in *Gli1-CreER*^*+*^*; Tm*^*fl/-*^ mice at 3 days/1 week/2 weeks/4 weeks after model generation. RFP^+^ cell% and RFP^+^/SOX9^+^ cell% were quantified in the **b** middle zone (MZ) and **c** posterior zone (PZ) of articular cartilage, respectively. *N* = 3–5. *P < 0.05, **P < 0.01, ****P < 0.000 1

After verifying *Gli1-CreER; Tm*^*fl/-*^ mice as feasible models for TMJ cartilage progenitor lineage chasing, we next performed ADD surgery in *Gli1-CreER; Tm*^*fl/−*^ mice 2 days after tamoxifen injection. The TMJ cartilage samples were collected at 3 days, 1 week, 2 weeks, and 4 weeks after model generation (Fig. S2). In the Sham mice, the RFP^+^ cell rate and RFP^+^/SOX9^+^ cell rate in the articular cartilage were gradually increased (Fig. 4f). Region-specific changes in the middle zone (mz) and posterior zone (pz) were found in ADD-TMJOA mice (Fig. 5a–c). In MZ, RFP^+^ lineage cells were increased at 3 days, and the cell rates reached the peak at 1 week, that 79% of the cells in articular cartilage are RFP^+^ lineages. In the meanwhile, there were only 20% of cells were RFP^+^ in the Sham group at this time point (Fig. 5b). Terminal deoxynucleotidyl transferase-mediated nick end labeling (TUNEL) staining showed that the cell apoptosis was sharply increased at 1 week and was sustained the increased apoptosis activity until 2 weeks (Fig. S3a, b). 5-ethynyl-2′-deoxyuridine (EdU) staining showed that the *Gli1* driven RFP^+^/EdU^+^ cell proportion in the cartilage of ADD mice was significantly higher than the Sham mice, signifying activated proliferation of FCSCs at the early stages of ADD-TMJOA (Fig. S3c, d). The proportion of RFP^+^/SOX9^+^ cells stepped up as well at the early stages and maximized at 2 weeks (Fig. 5b). These results indicated ADD-TMJOA induced the accelerated proliferation and differentiation of FCSCs in the middle zone of cartilage. Surprisingly, RFP^+^ cells and RFP^+^/SOX9^+^ cells did not show a significant difference between the Sham mice and ADD mice in the PZ of TMJ cartilage. These findings explained how ADD led to cartilage destruction through the functional changes of FCSCs. Interestingly, there were significantly increased SOX9^+^/RFP^-^ cells distributed in the ligament of PZ at 3 days in the ADD-TMJOA group. Then, the Aggrecan expression was markedly increased in the same area with increasing SOX9^+^/RFP^−^ cells in the PZ (Fig. 5c).

### *Sox9*^+^ cell lineage exhibits distinct expression and functional patterns in ADD-TMJOA cartilage and subchondral bone

**Fig. 6:**
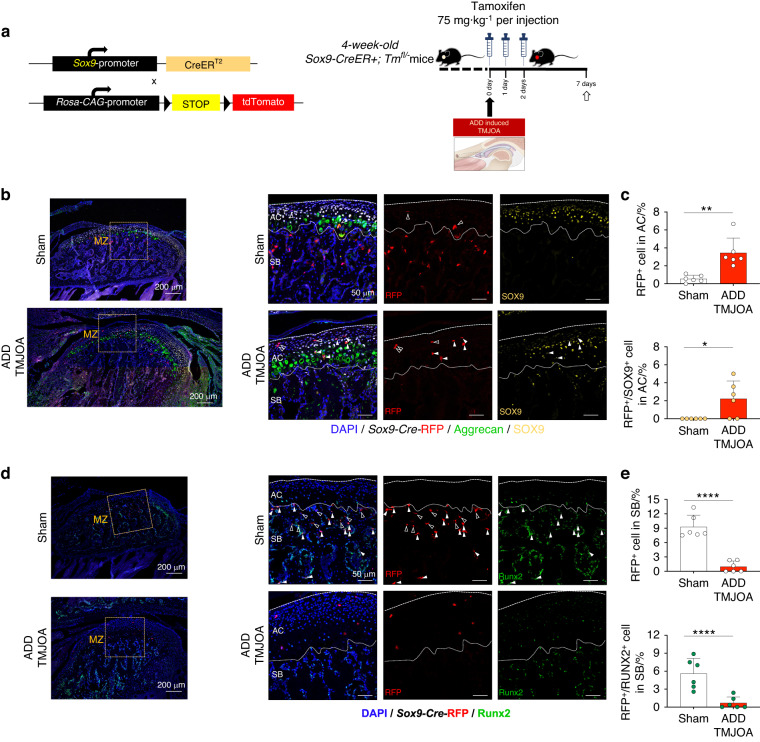
*Sox9*^+^ cell lineage exhibits distinct expression and functional patterns in ADD-TMJOA cartilage and subchondral bone. **a** Mating tactics for generating *Sox9-CreER*^*+*^*; Tm*^*fl/-*^ mice and strategies for generating the *Sox9-CreER*^*+*^*; Tm*^*fl/−*^ ADD-TMJOA mouse model. Tamoxifen injection was implemented right after ADD surgery, and mice were sacrificed at 1 week after ADD surgery. **b** Immunofluorescent staining of Aggrecan, SOX9, and RFP in the middle zone of TMJ cartilage in *Sox9-CreER*^*+*^*; Tm*^*fl/−*^ ADD-TMJOA mice. White solid arrows indicated RFP^+^/SOX9^+^ cells, and white hollow arrows indicated RFP^+^/SOX9^−^ cells. AC articular cartilage, SB subchondral bone. **c** Semi-quantification of RFP^+^ cell% and RFP^+^/SOX9^+^ cell% in the articular cartilage area. *N* = 6. **d** Immunofluorescent staining of RUNX2 and RFP in the middle zone of TMJ subchondral bone in *Sox9-CreER*^*+*^*; Tm*^*fl/-*^ ADD-TMJOA mice. White solid arrows indicated RFP^+^/RUNX2^+^ cells and white hollow arrows indicated RFP^+^/ RUNX2^−^ cells. AC articular cartilage, SB subchondral bone. **e** Semi-quantification of RFP^+^ cell% and RFP^+^/RUNX2^+^ cell% in the articular cartilage area. *N* = 6. *P < 0.05, **P < 0.01, ****P < 0.000 1

To clarify the origin of the SOX9^+^/RFP^-^ cells in the PZ with abnormally increased cartilage matrix expression, we used *Sox9-CreER; Tm*^*fl/-*^ mice to trace *Sox9*^+^ cell lineage. We activated *Sox9*^+^ linage right after ADD model generation and observed the expression and distribution of RFP^+^ cells in ADD TMJ (Fig. 6a). Both RFP^+^ cell rate and RFP^+^/SOX9^+^ cell rate were found significantly increase in the ADD-TMJOA group (Fig. 6b, c). We also activated *Sox9*^+^ linage by TM injection before ADD-TMJOA model generation (Fig. S4a), finding that the increment of both the RFP^+^ cell rate and RFP^+^/SOX9^+^ cell rate declined (Fig. S4b, c). These phenotypes indicated that there were a number of *Sox9*^+^ lineage cells activated by ADD-TMJOA. When *Sox9*^+^ linage cells were activated after ADD-TMJOA, we found that the transduction of RFP^+^ cells toward RUNX2^+^ osteoblastic lineage cells was also dramatically suppressed (Fig. 6d, e). However, the differentiation of *Sox9*^+^ lineage before ADD-TMJOA model generation was only slightly decreased in ADD group (Fig. S4d, e). These data indicate that there were *Sox9*^+^ chondrogenic progenitors formed in articular cartilage under ADD-TMJOA, while these *Sox9*^+^ lineage cells had abnormal functional changes, which cells fail to differentiate toward osteoblastic lineage and are incapable of the process of subchondral bone remodeling under OA condition.

### DR in ADD-TMJOA mice alleviates cartilage degeneration during TMJ growth

**Fig. 7:**
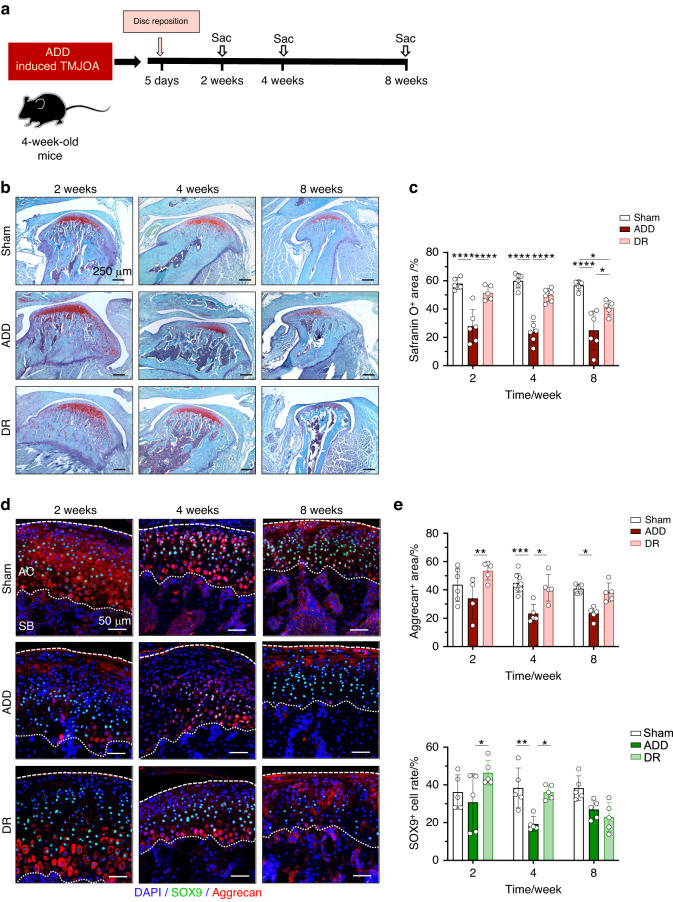
Disc reposition in ADD-TMJOA mice alleviated cartilage degeneration during TMJ growth. **a** Schematic diagram of Disc reduction (DR) treatment in ADD-TMJOA mouse. Disc reposition surgery was performed 5 days after ADD model generation. Mice were sacrificed at 2/4/8 weeks. **b** Safranin O staining of temporomandibular joint condylar cartilage at 2/4/8 weeks after ADD surgery. **c** Semi-quantification of safranin O^+^ area in ADD and DR mice. *N* = 4–8. **d** Aggrecan and SOX9 immunostaining of TMJ cartilage of the Sham nice, ADD-TMJOA mice, and DR mice at 2/4/8 weeks after ADD surgery. **e** Quantitative analysis of Aggrecan^+^ area and SOX9^+^ cell rate in the three groups. *N* = 4–8. *P < 0.05, **P < 0.01, ***P < 0.001, ****P < 0.000 1

**Fig. 8:**
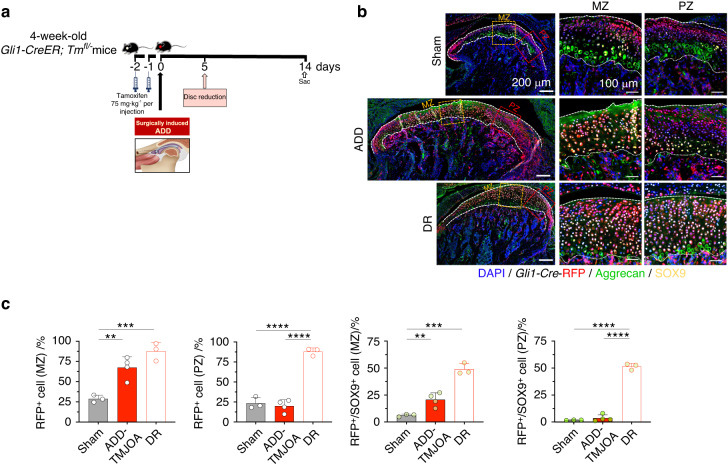
Disc reposition in ADD-TMJOA mice significantly stimulated the chondrogenic potential of *Gli1*^+^ FCSCs during TMJ growth. **a** Strategies for generating the DR mouse model using *Gli1-CreER*^*+*^*; Tm*^*fl/*^^−^ mice. **b** Expressions of *Gli1*-RFP, Aggrecan, and SOX9 were analyzed by immunofluorescent staining in TMJ cartilage in *Gli1-CreER*^*+*^*; Tm*^*fl/−*^ mice at 2 weeks after model generation. **c** RFP^+^ cell% and RFP^+^/SOX9^+^ cell% were semi-quantified in the middle zone (MZ) and posterior zone (PZ) of articular cartilage, respectively. *N* = 3–5. **P < 0.01, ***P < 0.001, ****P < 0.000 1

To further elucidate the pathophysiological role of FCSCs in cartilage repair and to explore potential treatment strategies, we designed a disc-repositioning model in the ADD-TMJOA mice (Fig. 7a). Three days after ADD, the disc repositioning surgery was performed (DR group), and samples were collected at different stages. Our pilot study showed that the Sham surgery in the ADD experiments and the Re-Sham surgery in the ADD/DR experiments didn’t lead to a statistical phenotypic difference of the TMJ cartilage and subchondral bone (Fig. S5a, b). The success of disc repositioning was judged by the restored position of the disc by Safranin O staining (Fig. 7b), and the repositioning was considered successful if the thinner middle part of the articular disc was located above the top of the condyle. In all, 75%, 50% and 75% of discs were successfully repositioned in the 2-week, 4-week, and 8-week DR groups, respectively. The Safranin O^+^ matrix secretion area of DR mice was significantly recovered within 2 weeks (Fig. 7c). The Aggrecan expression area SOX9^+^ cell rate was found to be identical in the DR group and the Sham group and was significantly higher than the ADD group (Fig. 7d, e). These results demonstrated that the homeostasis of chondrogenic differentiation and cartilage matrix secretion was re-established when disc displacement was released at the early stage in ADD mice. *Gli1-CreER*^*+*^*; Tm*^*fl/−*^ mice were used to further validate the potential role of FCSCs in the recovery of impaired chondrocytes (Fig. 8a). In the DR group, the RFP^+^ cell proliferation activity was also recovered in both MZ and PZ at 2 weeks DR group (Fig. 8b, c). Notably, the RFP^+^/SOX9^+^ cell rate was dramatically increased to almost 50% of the whole cells in both zones, which was significantly higher than the Sham mice and the ADD groups (Fig. 8b, c), suggesting the vanishment of the pathological change of FCSCs fate with spatial heterogeneity induced by ADD-TMJOA. The capacity of FCSCs was actively stimulated to restore chondrogenic differentiation and produce a cartilage matrix when the disc was repositioned.

**Fig. 9:**
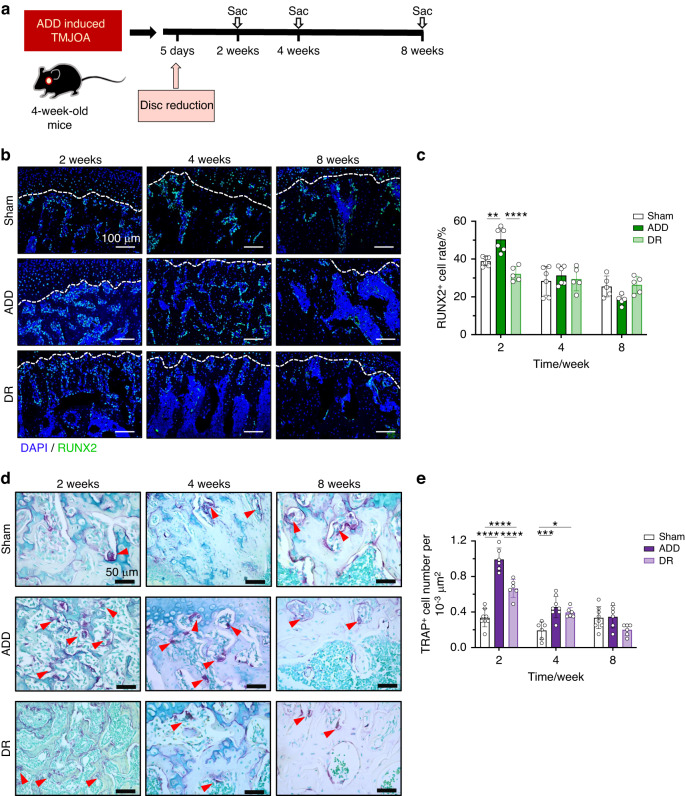
Disc reposition alleviated TMJ subchondral bone destruction and promoted condylar growth in ADD-TMJOA mice. **a** Schematic diagram of Disc reduction (DR) in ADD-TMJOA mouse. **b** Immunofluorescent staining of RUNX2 expressions in TMJ subchondral bones in DR mice at 2/4/8 weeks after ADD surgery. **c** Semi-quantification of RUNX2^+^ cell% in subchondral bone area. *N* = 5–6. **d** TRAP staining in the subchondral bone area of DR mice at 2/4/8 weeks after ADD surgery. **e** Semi-quantification of TRAP^+^ cell% in TMJ subchondral bone area. *N* = 6–8. *P < 0.05, **P < 0.01, ***P < 0.001, ****P < 0.000 1

Next, we investigated the subchordal bone remodeling after disc repositioning (Fig. 9a). At 2 weeks, the RUNX2^+^ cell rate in the DR group was statistically lower than the ADD group (Fig. 9b, c). TRAP^+^ cell numbers were increased compared to the Sham group but were significantly less than the ADD mice (Fig. 9d, e). There was no difference in RUNX2^+^ cell rate or TRAP^+^ cell numbers between the 3 groups at 8 weeks (Fig. 9c, e). Through Micro CT scanning, we found that the condylar morphology in the DR mice was similar to the Sham group (Fig. S6a). In comparison with the ADD-TMJOA group, the RH of the DR mice was increased, and the CW was decreased at the late stage. However, the CL was not different within the 3 groups (Fig. S6b). In addition, microCT measurements showed that the phenotype of bone loss and osteoporosis in ADD mice were largely alleviated by DR. (Fig. S6c). The results suggested that disc repositioning surgery prevents subchondral bone destruction, which may be beneficial for rebuilding the growth homeostasis of the mandible.

Finally, we followed the condylar changes in adolescent patients with unilateral ADD without reduction (ADDWoR). These young patients were found to manifest with facial asymmetry malformation, deviation of the midline of lower teeth, as well a shortened condylar head examined by cone bean CT (Fig. S7a, b, Table 2). The volume and height of the TMJ condyle on ADD affected side were also significantly reduced (Fig. S7c). Nine to twelve months after disc repositioning surgical treatment, while CL was not significantly changed, the condylar volume and condylar height were both remarkably increased (Fig. S7d). Thus, the condyle of these adolescent patients and growing mice share the same alteration under ADD-TMJOA or disc repositioning.

**Table 2:** The general information of ADDWoR adolescent patients

Patients	Gender	Age	Diagnosis	Symptoms	Surgical interventions
Patient 1	Female	14	Left ADDWoR, Left condylar resorption	Limited mouth opening, pain	Disc repositioning surgery (mini-screw anchor) of left TMJ
Patient 2	Female	16	Left ADDWoR, Left condylar resorption	Pain	Disc repositioning surgery (mini-screw anchor) of left TMJ
Patient 3	Female	16	Left ADDWoR, Left condylar resorption	Limited mouth opening, pain	Disc repositioning surgery (mini-screw anchor) of left TMJ
Patient 4	Female	18	Right ADDWoR, Right condylar resorption	Limited mouth opening, pain	Disc repositioning surgery (mini-screw anchor) of right TMJ
Patient 5	Female	13	Right ADDWoR, Right condylar resorption	Mouth skewed, pain	Disc repositioning surgery (mini-screw anchor) of right TMJ
Patient 6	Male	17	Right ADDWoR, Right condylar resorption	Limited mouth opening, pain	Disc repositioning surgery (mini-screw anchor) of right TMJ

## Discussion

In previous studies, condylar height loss and secondary mandibular growth disorder were found in adolescent ADDWoR patients.^32–34^ However, the underlying cellular mechanism that may give rise to the TMJ cartilage growth retardation was unclear over time. Therefore, our study identified the correlation between the fate changes of chondrogenic progenitor lineages and the OA degradation process during TMJ cartilage growth. In this study, we, for the first time, generated an ADD-induced TMJOA mouse model and chased the FCSCs lineages under abnormal biological stress. We demonstrated that the degeneration of cartilage and subchondral bone were correlated with FCSCs lineage fate changes. *Gli1*^+^ FCSCs contributed to cartilage injury and remodeling by enhancing their proliferation and chondrogenic differentiation with spatial heterogeneity at the early stage yet lost their normal functions and finally led to cartilage destruction at late stages. A previous study using finite element analysis demonstrated that disc displacement induced an increase of pressure in the disc and frictional coefficients on the condyle,^35^ thus a possible explanation for the correlation between condylar height and disc morphological changes could attribute to the condylar motion squeezes the disc forward, increases stress on the surfaces of condyle cartilage, which results in the function loss of FCSCs. Then TMJOA initiates, and the condylar height and disc length were consequently decreased.

The TMJ cartilage consists of various proportions of both fibrous and cartilaginous tissue. Fibrocartilage not only provides tensile and compressive strength but also stores FCSCs to serve as critical cell resources for TMJ development and homeostasis.^25,28^ The TMJ surface is coated with fibrocartilage instead of hyaline cartilage, so the pathological process of cartilage degeneration in TMJOA is distinct from other joints. When the TMJ disc is anteriorly displaced, the fibrocartilage in the joint surface is especially vulnerable to OA inflammatory damage. Once the fibrocartilage is damaged, it causes permanent tissue loss and disability, including limitation of mandibular function and growth.^7^ Thus, preserving the regenerative capabilities of FCSCs in fibrocartilage could potentially be an effective targeted therapy strategy for ADD cartilage repair. One intriguing phenotype observed in the TMJ cartilage under ADD is the regional specificity of chondrocyte fate changes in the middle zone and posterior zone. One possible reason is that there reside other chondrogenic progenitors in addition to the *Gli1*^+^ FCSCs lineage in the fibrous connective tissue attached to the different cartilage zones. For instance, previous studies have shown that there are resided Scx^Lin^ cells in the tendon around the condyle that directly form a subset of chondrocytes in TMJ.^36^ The diversity of chondrogenic progenitor sources may contribute to the phenotypic difference of different zones in TMJ cartilage under ADD. We found that the chondrogenesis transduction of Sox9^+^ cells was markedly blocked by ADD, signifying the loss of differentiation capacity of these chondrogenic progenitor cells. These findings hint to us that the abnormal disc-condylar structure would not only affect the FCSCs in the fibrocartilage surface but consistently affect the expression and functional features of FCSCs lineages even in the deeper area, including in subchondral bones.

In our study, both the DR mouse model and adolescent ADDWoR patients with DR surgery were found with significant recovery of condylar height loss. These phenotypes provide evidence that restoration of the balance of disc-condyle structure could alleviate cartilage degeneration, which supports the treatment strategy to recover normal disc position. Our animal model also showed that the TMJ disc turned out to be perforated at later stages of ADD-TMJOA. After disc perforation, the destruction of TMJ cartilage is aggravated, which is possibly due to further impairment of chondrogenic progenitor functions under the more severe inflammatory environment in the joint. On the other hand, ADD at early stages activated both the subchondral bone resorption and formation, which demonstrates the indispensability of mechanical forces loading for bone remodeling.^37^ However, the abnormal mechanical stress by ADD could not sustain a balanced bone remodeling at later stages, and subchondral bone loss eventually occurs. These results remind us of the importance of releasing disc displacement at the early stage, aiming at promoting residual FCSCs chondrogenic capacity, preserving subchondral bone homeostasis, and improving condylar development for adolescent patients.

As a surgically induced disc displacement mouse model, our model could not fully mimic the realistic pathological changes of ADD patients. The anatomic difference between rodents and humans, as well as the different duration of disc displacement from initiation to observation between our model and most patients, bring some limits to the strength of our observations. A longer-term observation period, as well as other primate animal models, may be used to further verify our findings in the future.

## Conclusion

To the best of our knowledge, this is the first study that investigates the functional roles of FCSCs in TMJ metabolism under disc displacement. This study reveals the importance of the *Gli1*^+^ FCSCs transduction toward *Sox9*^+^ chondrogenesis lineage in mediating chondrocyte homeostasis and subchondral bone remodeling. DR could preserve FCSC capacity as a chondrogenic stem cell resource that contributes to cartilage growth and repair. Taken together, our study supports the treatment strategy to recover normal disc position, aimed at promoting FCSCs chondrogenic capacity and improved condylar development for adolescent patients.

## Materials and methods

### Ethics approval information

This study was performed in strict accordance with the recommendations in the Guide for the Care and Use of Laboratory Animals of Sichuan University. Animal procedures were performed according to protocols approved by the Animal Ethics Committee of Sichuan University (WCHSIRB-D-2020-476). All animal experiments followed the Animal Research: Reporting of In Vivo Experiments (ARRIVE) guidelines. For collecting patient medical images, the study was approved by the institutional review board of West China Hospital of Stomatology, Sichuan University (WCHSIRB-D-2020-116), and all patients signed the informed consent. Patients or the public were not involved in the design, conduct, reporting, or dissemination plans of our research.

### Power calculation

The number of mice per experimental group was based on a power calculation using data from our preliminary experiments. We found that a sample size of 5 mice per group would provide greater than 80% power to detect at least a 50% difference between ADD and Sham groups in RFP^+^ cell numbers in *Gli1-CreERT*^*+*^*; Tm*^*fl/−*^ mice. To account for the possibility of a loss of mice due to death before study completion, we used an N of 5–8 for RFP^+^ cell number counting.

### Animals

C57BL/6 wild-type male mice were purchased from Dashuo Experimental Animal Laboratories (Chengdu). Gli1^tm3(creERT2)Alj^/J mice (JAX#007913, abbreviated *Gli1-CreER*^*+*^), Tg(Sox9-cre/ERT2)1Msan/J mice (JAX#018829, abbreviated *Sox9-CreER*^*+*^) and B6.Cg-Gt(ROSA)26Sor^tm14(CAG-tdTomato)Haze^/J mice (JAX#07908, abbreviated *Tm*^*fl/fl*^) were obtained from the Jackson Laboratory. Offspring were genotyped at 2 weeks old and conducted experiments at 4 weeks old. To generate tdTomato-conditionally activated mice, *Tm*^*fl/fl*^ mice were crossed with *Gli1-CreER*^*+*^ mice and *Sox9-CreER*^*+*^ mice. Animals were housed in the Experimental Animal Center of the West China Hospital with free access to water and food at 25 °C with 40% humidity under a 12 h light/dark cycle. For ADD animal model and DR animal model generations described below, confounders between different groups were not controlled.

### Generation of a mouse model with surgically induced ADD

The surgical method of ADD mouse model was modified based on previous studies described for rats and rabbits.^38–40^ Mice were anesthetized using i.p. injection with tribromoethanol at 0.12 μL·g^−1^ body weight (T903147, Macklin, China). The right preauricular region, as the surgical region, was shaved and sterilized with iodine and 75% ethanol and followed by local anesthesia with subcutaneous injection of 0.1 ml lidocaine hydrochloride (2%). A curved incision at approximately 1 cm in diameter was made parallel to the ears, extending from the temporalis muscle to the inferior edge of the masseter muscle. The overlying parotid gland was carefully exposed and dissected, and the superficial temporal vein was electro-coagulated. The zygomatic arch and TMJ capsule were exposed. The periosteum of the zygomatic arch was separated forward to the lower orbital margin and backward to expose the zygo-temporal suture. The lateral joint capsule was opened by a tweezer, the medial attachment was retained, and the articular disc and the lower condyle were exposed. A hole at 1 mm diameter was carefully drilled anterior to the front junction of the zygomatic arch using a 0.3 mm round bur. A round needle with a 6-0 nylon suture attached crossed the hole and was tied, then penetrated through the anterior band of the disc. The disc was pulled forward by the nylon suture. After confirming the disc was at the front of the condylar head, the suture was double-tied to the prepared hole in the zygomatic arch. After disc displacement, the wound was thoroughly irrigated with saline, and the anterior position of the disc was verified by about 1 mm deviation of the lower midline of teeth to the non-operative side. Finally, the wound was sutured in layers. Mice in the Sham group went through the same surgical procedure, including exposing the joint capsule, except drilling the hole in the zygomatic arch and pulling the disc forward. After ADD/Sham surgery, soft food was provided, and normal chow was provided one week later.

### DR in ADD mouse model

ADD mice were randomly selected for disc repositioning surgery 5 days after the initial ADD surgery. After anesthesia by i.p. injection with tribromoethanol at 0.12 μL·g^−1^ body weight (T903147, Macklin, China), another incision was made along the previous incision, the TMJ capsule was exposed. The 6-0 nylon suture was carefully moved with the intact disc preserved. Then the displaced disc was released from adhesion and was pulled back on the top of the condyle. The muscle and skin were sutured. The re-Sham mice went through the second operation that exposed the TMJ capsule again without changing the disc position. The same postoperative care as initial surgery was provided.

### Specimen preparation

Mice in ADD and Sham groups were randomly chosen and were sacrificed at 1, 2, 4, and 8 weeks after model generation. Mice in DR and re-Sham group were sacrificed at 2, 4, and 8 weeks after ADD surgery. After anesthetizing with isoflurane, 25 mL ice-cold heparin PBS (10 U·mL^−1^ heparin sodium in 0.01 mol·L^−1^ PBS) was injected transcardially. Next, 25 mL 4% PFA (4% paraformaldehyde in 0.01 mol·L^−1^ PBS, pH 7.4) was infused transcardially for tissue fixation. The intact TMJs in different groups were dissected and fixed at 4 °C for 24 h. For decalcification, Specimens were decalcified with 15% EDTA (15% ethylenediaminetetraacetic acid disodium salt solution, pH 7.5) for 7–21 days at 4 °C. Then the TMJ specimens were paraffin-embedded sagittally and were consecutively cut at 5 μm. For *Gli1-CreER*^*+*^ and *Tm*^*fl/fl*^ mouse, the decalcified samples were embedded and frozen in optimum cutting temperature compound (OCT) after dehydrating by 30% sucrose solution overnight and cut into 6 μm thick sections.

### Micro CT

Samples at 2, 4, and 8 weeks were selected for Micro CT scanning (Scanco, Switzerland) at 70 kVp, 200 µA with a spatial resolution of 7 µm. Scanco Evaluation software was used for bone analysis and three-dimensional reconstruction. Three cubes in size of 210 × 210 × 210 µm^3^ were selected in the anterior, middle, and posterior subchondral bone regions. 3D reconstruction of the mandible was performed to measure CL, width, and RH.^41^ The RH is measured by the vertical distance from the sigmoid notch to the midpoint of the condyle.

### Histology and modified Mankin score

Hematoxylin and eosin staining (H&E, Biosharp, China) and Safranin O/ fast green (SO) staining (SO&FG, Solarbio, China) were performed according to the manufacturer’s instructions. The number of chondrocytes and thickness of cartilage were counted using H&E staining sections. The thickness of condylar cartilage was assessed three times at quarter points of the anterior, middle, and posterior cartilage in each region. The average thickness of cartilage was calculated as the mean value of nine data from each TMJ sample and ultimately used for statistical analysis (Fig. S8). The Safranin O^+^ area was calculated using ImageJ 1.51. Modified Markins score was used to assess the severity of cartilage arthritis, based on the number and morphology of chondrocytes, the safranin O positive region of cartilage matrix, and cartilage integrity, normal cartilage is scored 0-1 point. Totally, 2–4 points are assigned to mild OA. The Image 6.0 image analysis system (Media Cybernetics, Rockville, MD) was used for semiquantitative analysis.

### Immunofluorescent staining

For immunofluorescent staining, the following antibodies were used: Aggrecan (1:100, 969D4011, Invitrogen), Collagen II (1:50, SC-52658, Santa Curz), SOX9 (1:200, ab185966, Abcam), RUNX2 (1:100, ET1612-47, Huaan). The secondary antibodies included goat anti-rabbit Alexa Fluor 488/ 568/ 647, anti-mouse Alexa 488/ 568 (1:500, Invitrogen). Apoptotic cells were detected by TUNEL staining (In Situ Cell Death Detection Kit, 11684795910, Roche’s) according to the recommended protocol. Slices were detected by laser scanning confocal microscopy (FV3000, Olympus).

Aggrecan^+^ and Collagen II^+^ area was calculated using ImageJ software. Three square frames (0.12 mm × 0.12 mm) in cartilage or subchondral bone were randomly selected to calculate the numbers of SOX9^+^ cells, RUNX2^+^ cells, and TUNEL^+^ cells.

### Tartrate-resistant acid phosphatase (TRAP) staining

TRAP staining was conducted to detect osteoclasts based on the TRAP staining kit instruction (NO387, Sigma). Three regions of interest (0.15 mm × 0.15 mm) were selected from the subchondral bone in the anterior, middle, and posterior regions of the condyle. Purplish red cells containing more than one nucleus were counted into osteoclasts.

### RNA sequencing analysis

The mRNA levels were detected by RNA sequencing. Fresh cartilage in condyles of 1w Sham group and ADD group mice were carefully removed and cleaned with 4°enzyme-free PBS. The cartilage tissues were removed completely and sent for analysis as far as possible. After Total RNA extraction by Trizol reagent (Thermofisher, 15596018), RNA was purified and reverse-transcribed to create the cDNA library that was sequenced and run with Illumina NovaseqATM 6000 sequence platform. Genes differential expression analysis was performed by DESeq2 software between two different groups and by edgeR between two samples. The genes with the parameter of false discovery rate (FDR) below 0.05 and absolute fold change ≥ 1.5 were considered differentially expressed genes.

### Tamoxifen administration

Tamoxifen (T5648, Sigma) was suspended in corn oil (S50856, Yuanye) at 10 mg·mL^−1^. In the ADD and DR model generation experiment, *Gli1-CreER*^*+*^*; Tm*^*fl/-*^ mice were injected intraperitoneally with tamoxifen 2 days before model generation (75 mg·kg^−1^ body weight per day, for 2 consecutive days). *Sox9-CreER*^*+*^*; Tm*^*fl/−*^ mice were injected intraperitoneally with tamoxifen 7 days before model generation (75 mg·kg^−1^ body weight per day, for 3 consecutive days).

### EdU assay

EdU (30 mg·kg^−1^) (A10044, Invitrogen) was injected i.p. 24 hours prior to sacrifice. EdU^+^ cells were detected with the Cell-Light^TM^ EdU Apollo 488 In Vitro Kit (C10310-3, Riobobio) according to the recommended protocol.

### Patients and study design

A retrospective study included 12 condyles (6 normal and 6 with ADDwoR) from adolescent patients with unilateral ADDwoR treated by disc repositioning surgery at West China Hospital of Stomatology, Sichuan University, was conducted from April 2021 to August 2022.

Inclusion criteria were (1) ≤18 years old; (2) diagnosed with unilateral ADD without reduction; (3) treated by disc repositioning surgery; (4) with complete preoperative and postoperative Cone-beam CT (CBCT) data of both TMJ.

Patients with syndromes, cleft palates, orthodontics treatment, and orthognathic surgery history were excluded. ADDwoR patients were diagnosed by experienced doctors according to the diagnostic criteria for temporomandibular disorders.

### Imaging

CBCT data were collected 2–4 weeks before and 6–12 months after surgery. The scanning speed of the CBCT was 26.3 s, with 120 kV and 20 mA, and each layer thickness was set as 0.16 mm. Patients were in a seated position with the orbital-ear plane parallel to the horizon and asked to keep the eyes forward, intercuspation, and tongue position during the examination.

### 3D image measurements

The scan data were stored in the format of “.dicom”, which was imported into and constructed virtual 3D surface models of the anatomic condylar structures of interest with Mimics 21 software (Materialize, NV, Belgium). The 3D measurement started from condylar image segmentation and reconstruction based on previous studies.^42,43^ The superior contour of the condyle was determined as the first radiopaque point found in the upper articular area. The lowest point of the sigmoid notch is the marker point of the bottom of the condyle. The 3D condylar image was reconstructed after the isolation, and the condylar volume was measured. Condylar height was measured as the vertical distance from the apex of the condyle to the largest cross-section of the reconstructed condyle in the sagittal view, and CL was analyzed as the distance between the most anterior and posterior points of the condylar head.

### Statistical analysis

The mean and standard deviation of data were analyzed by GraphPad Prism 9 analysis software. Two-way analysis of variance (ANOVA) was used to compare the data of the two groups at multiple time points, a *P* value < 0.05 was considered to be statistically significant. The staining results were analyzed by ImageJ 1.51 (Leeds Precision Instruments, USA) by an individual analyzer using blind sections.

### Supplementary information

Supplementary materials
