# Supplementary material for: Divergent chondro/osteogenic transduction laws of fibrocartilage stem cell drive temporomandibular joint osteoarthritis in growing mice
Source: Int J Oral Sci. 2023 Aug 25;15:36. doi: 10.1038/s41368-023-00240-5 (PMC10457315; doi:10.1038/s41368-023-00240-5)
Supplement: Supplementary file 1 — Supplementary materials [file 41368_2023_240_MOESM1_ESM.docx]

***Divergent chondro/osteogenic transduction laws of fibrocartilage stem cell drive temporomandibular joint osteoarthritis in growing mice***

***Supplementary Materials***

Ruiye Bi^1#^, DDS/PhD; Qianli Li^1#^, DDS; Haohan Li^1^, DDS, Peng Wang^1^, DDS/PhD; Han Fang^1^, DDS; Xianni Yang^1^, DDS; Yiru Wang^1^, DDS; Yi Hou^2^, PhD; Binbin Ying^3^, DDS/PhD; Songsong Zhu^1*^, DDS/PhD

^1^State Key Laboratory of Oral Diseases, National Clinical Research Center for Oral Diseases, Department of Orthognathic and TMJ Surgery, West China Hospital of Stomatology, Sichuan University, Chengdu, 610041, China

^2^State Key Laboratory of Oral Diseases, National Clinical Research Center for Oral Diseases, West China Hospital of Stomatology, Sichuan University, Chengdu, 610041, China

^3^Department of Stomatology, Ningbo First Hospital, Ningbo 315000, China

*** Corresponding author:** Songsong Zhu,

E-mail: [ZSS_1977@163.com](mailto:ZSS_1977@163.com); Postal address: Department of Orthognathic and TMJ Surgery, No. 14, 3^rd^ Section of Ren Min Nan Rd, West China Hospital of Stomatology, Sichuan University, Chengdu, 610041, China

**# These authors contribute equally to this work**

**
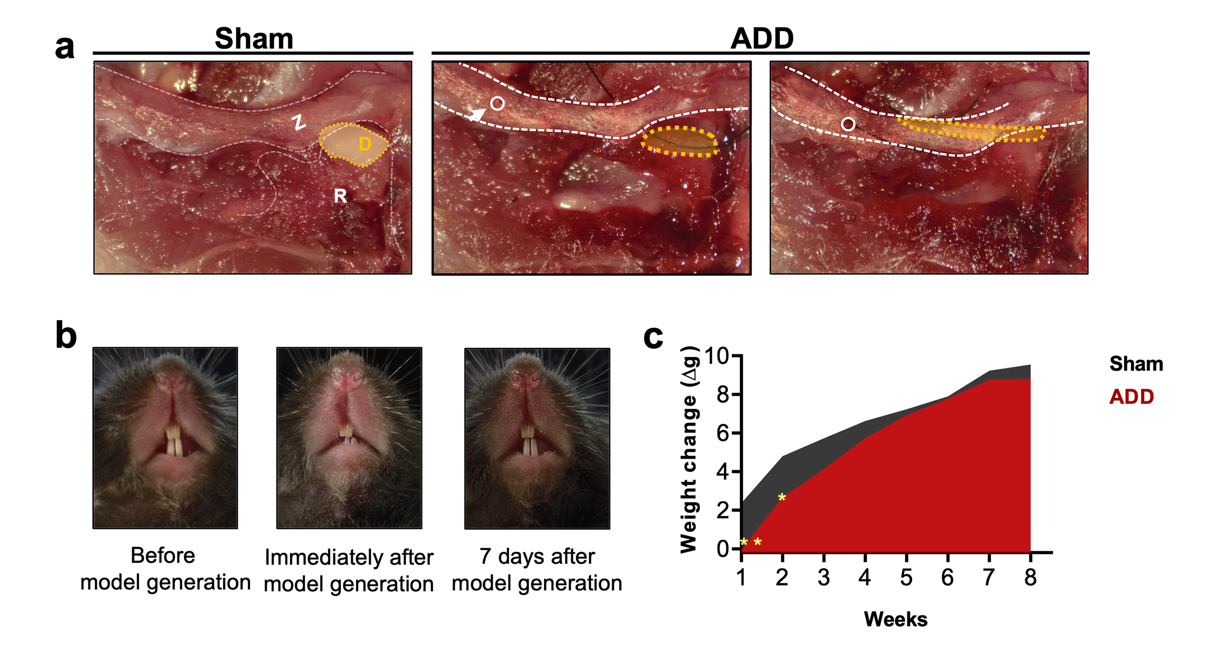
**

**Figure S1:** (a) Anatomical images of Sham and ADD surgery process. In the Sham group, the zygomatic arch was exposed without releasing the disc attachment, then the incision was sutured. In the ADD group, a small hole at 0.3 mm diameter was drilled in the anterior area of the zygomatic arch, then the articular disc was released, being penetrated with a 6-0 nylon suture. the disc was pulled forward, and was fixed to the prepared hole. (b) Frontal views of the anterior bite in ADD mouse model before ADD surgery, immediately after ADD surgery and 7 days after ADD surgery. (c) The body weight changes of Sham and ADD mice from 1 week to 8 weeks after ADD surgery. N=4-5. *P < 0.05, **P < 0.01.


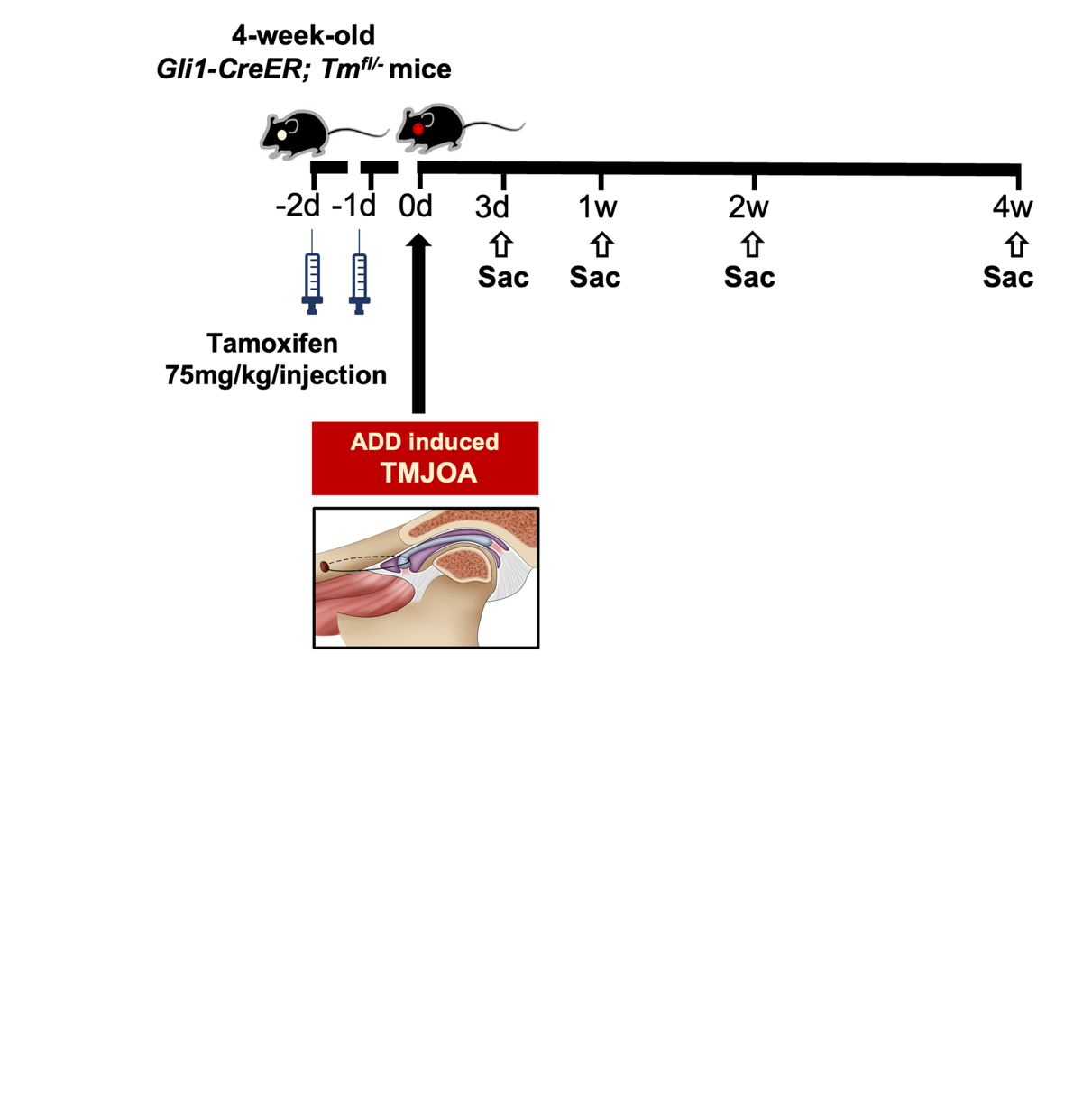
**Figure S2:** Strategies for generating the ADD-TMJOA mouse model using *Gli1-CreER^+^; Tm^fl/-^* mice.

**
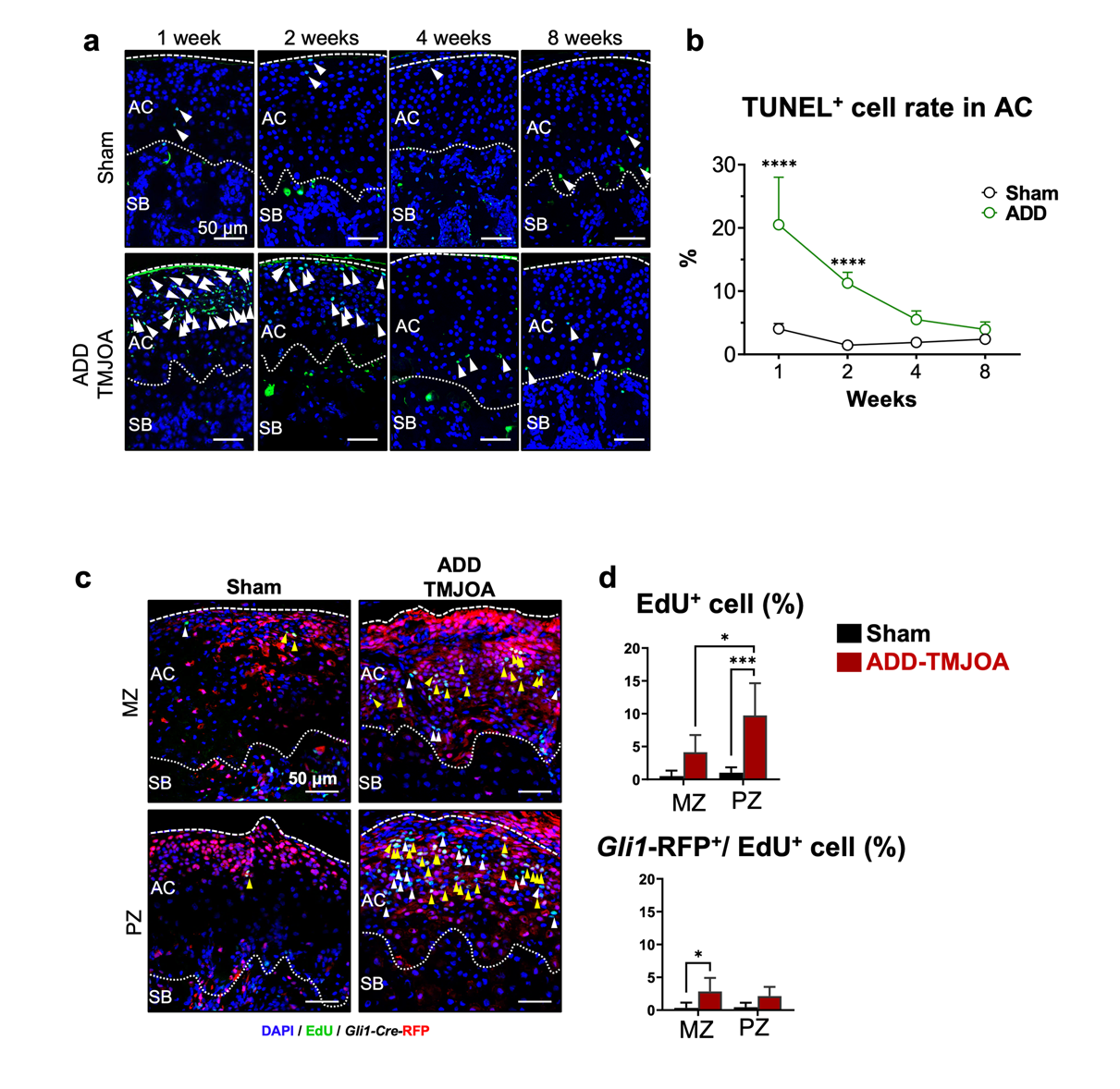
**

**Figure S3:** (a) TUNEL staining of TMJ articular cartilage at 1/2/4/8 weeks after ADD surgery. (b) TUNEL^+^ cell% in the articular cartilage area was semi-quantified. White arrows indicated TUNEL^+^ cells. N=6-7. (c) EdU staining of TMJ cartilage in *Gli1-CreER^+^; Tm^fl/-^* mice at 1 week after ADD surgery. MZ: middle zone of the condylar cartilage. PZ: posterior zone of the condylar cartilage. White arrows indicated the RFP^-^/EdU^+^ cells, yellow arrows indicated the RFP^+^/EdU^+^ cells. (d) EdU^+^ cells% and *Gli1*-RFP^+^/EdU^+^ cells in the TMJ articular cartilage area were semi-quantified. *P < 0.05, ***P < 0.001.

**
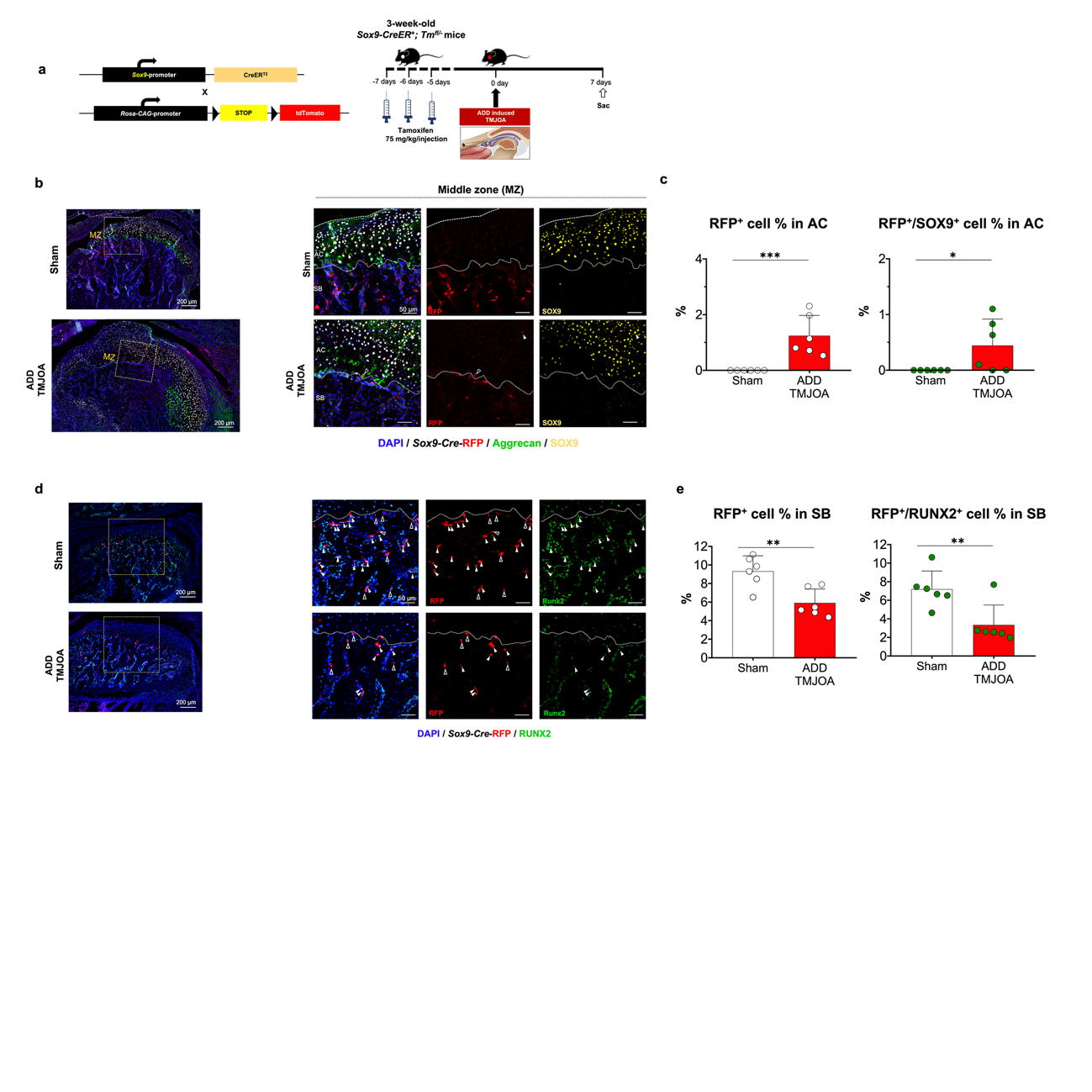
Figure S4:** (a) Mating tactics for generating *Sox9-CreER^+^; Tm^fl/-^* mice and strategies for generating the *Sox9-CreER^+^; Tm^fl/-^* ADD-TMJOA mouse model. Surgery was performed at 1 week after tamoxifen injection and mice were sacrificed at 1 week after ADD surgery. (b) Immunofluorescent staining of Aggrecan, SOX9 and RFP in the middle zone of TMJ cartilage in *Sox9-CreER^+^; Tm^fl/-^* ADD-TMJOA mice. White solid arrows indicated RFP^+^/Sox9^+^ cells and white hollow arrows indicated RFP^+^/SOX9^-^ cells. AC: articular cartilage, SB: subchondral bone. (c) Semi-quantification of RFP^+^ cell% and RFP^+^/SOX9^+^ cell% in the articular cartilage area. N=6. (d) Immunofluorescent staining of RUNX2 and RFP in the middle zone of TMJ subchondral bone in *Sox9-CreER^+^; Tm^fl/-^* ADD-TMJOA mice. White solid arrows indicated RFP^+^/RUNX2^+^ cells and white hollow arrows indicated RFP^+^/ RUNX2^-^ cells. AC: articular cartilage, SB: subchondral bone. (e) Semi-quantification of RFP^+^ cell% and RFP^+^/RUNX2^+^ cell% in the subchondral bone area. N=6. *P < 0.05, **P < 0.01, ***P < 0.001.


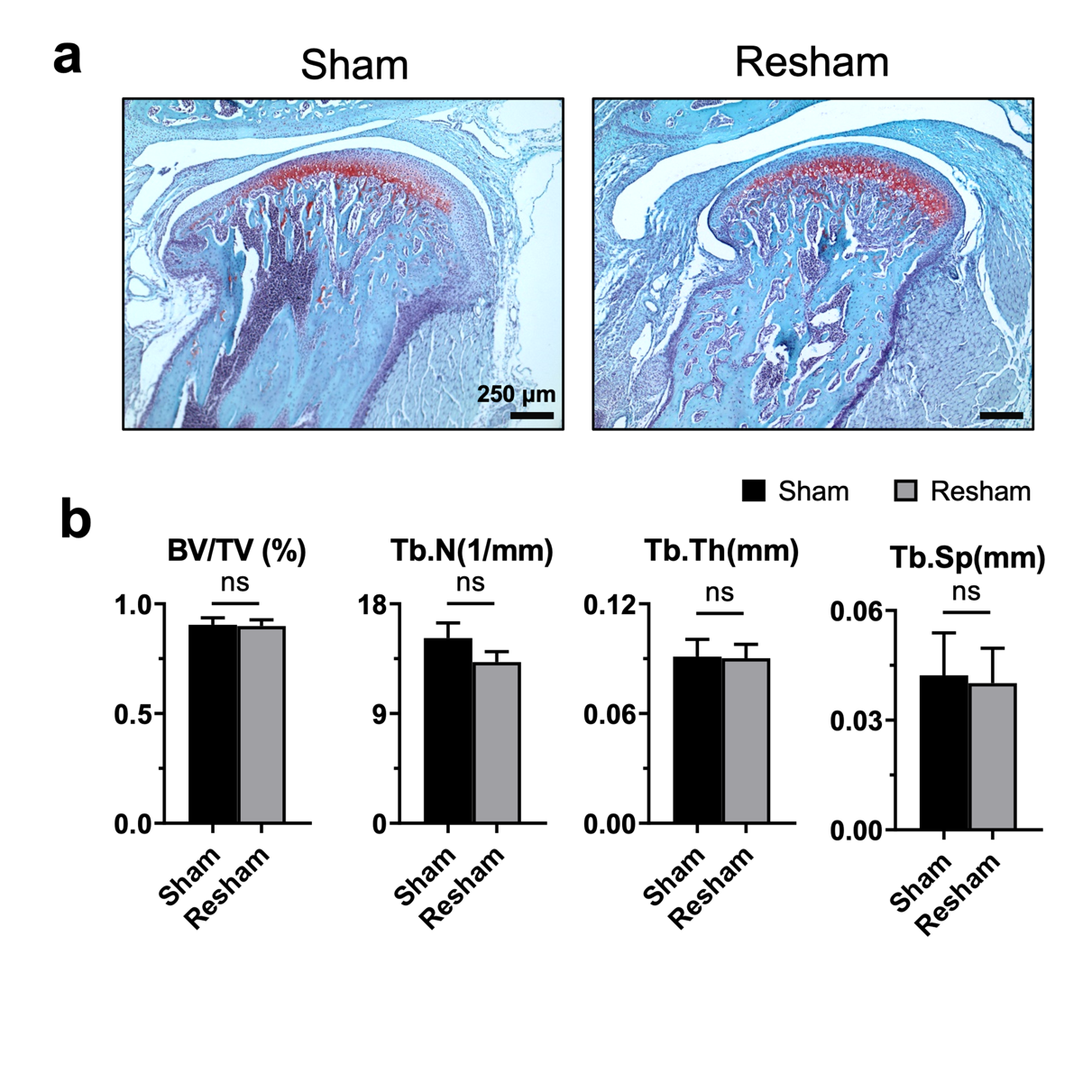


**Figure S5:** (a) Safranin O staining of TMJ cartilage in Sham mice and Resham mice; (b) Micro CT analyses of subchondral bone in Sham mice and Resham mice.

**Figure S6:** (a)
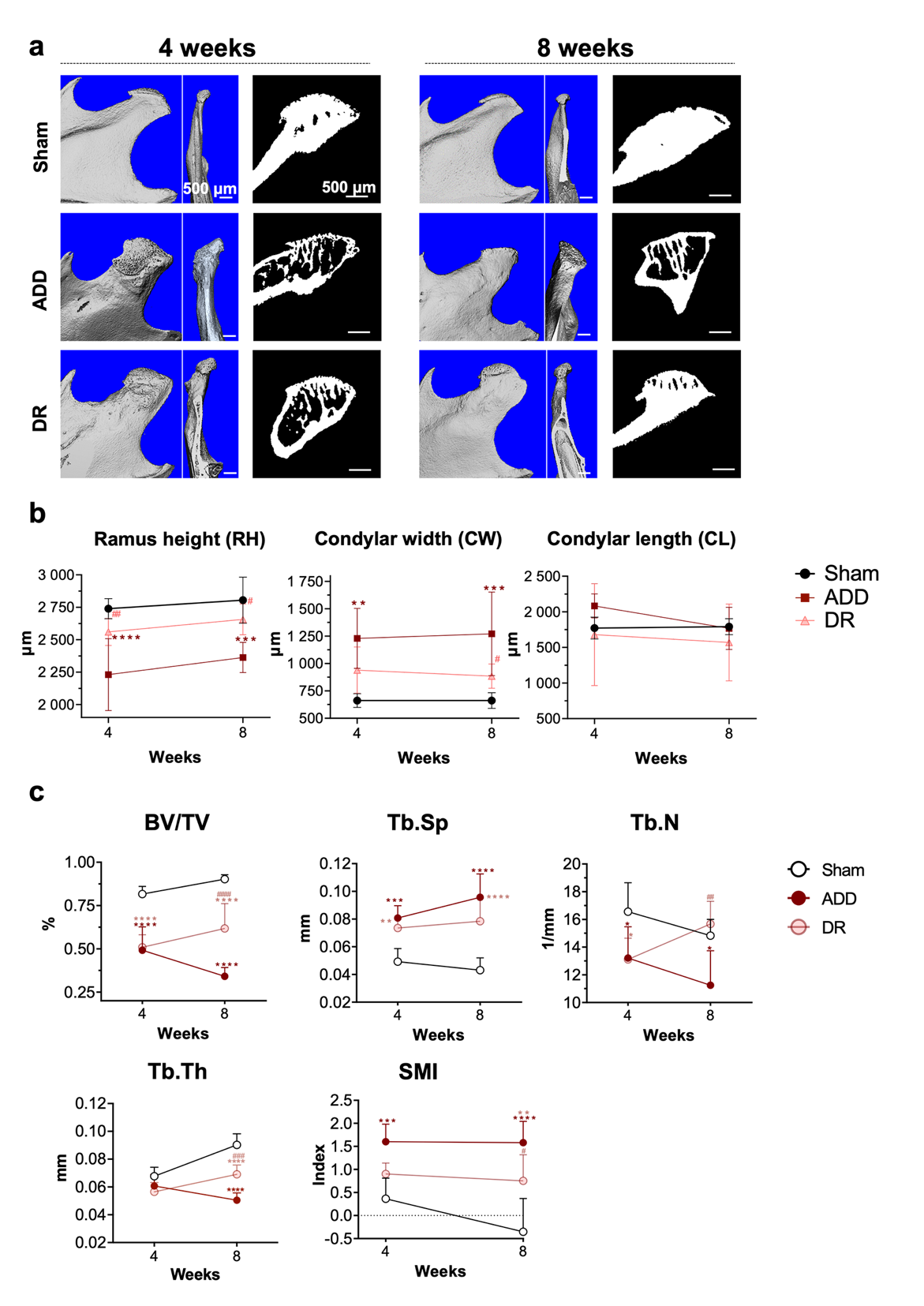
Representative images of Micro CT analysis of condylar bones of Sham, ADD-TMJOA and DR mice groups at 4/8 weeks after ADD surgery. (b) The ramus height (RH), condylar width (CW), and condylar length (CL) were analyzed. N=5-7. Compared with Sham group, **p<0.01, ***p<0.001, ****p<0.0001; compared with the ADD group, ^#^p<0.05, ^##^p<0.01. (c) The quantification analysis of ROI. BV/TV(%): bone volume fraction; Tb.Sp (mm): trabecular separation; Tb.N (1/mm): trabecular number; Tb.Th (mm): trabecular thickness; SMI: structure model index. N=6-7. Horizontal line and error bars indicate mean ± SD. *p<0.05, ***p<0.001, ****p<0.0001; compared with the ADD group, ^#^p<0.05, ^##^p<0.01, ^###^p<0.001, ^####^p<0.0001.


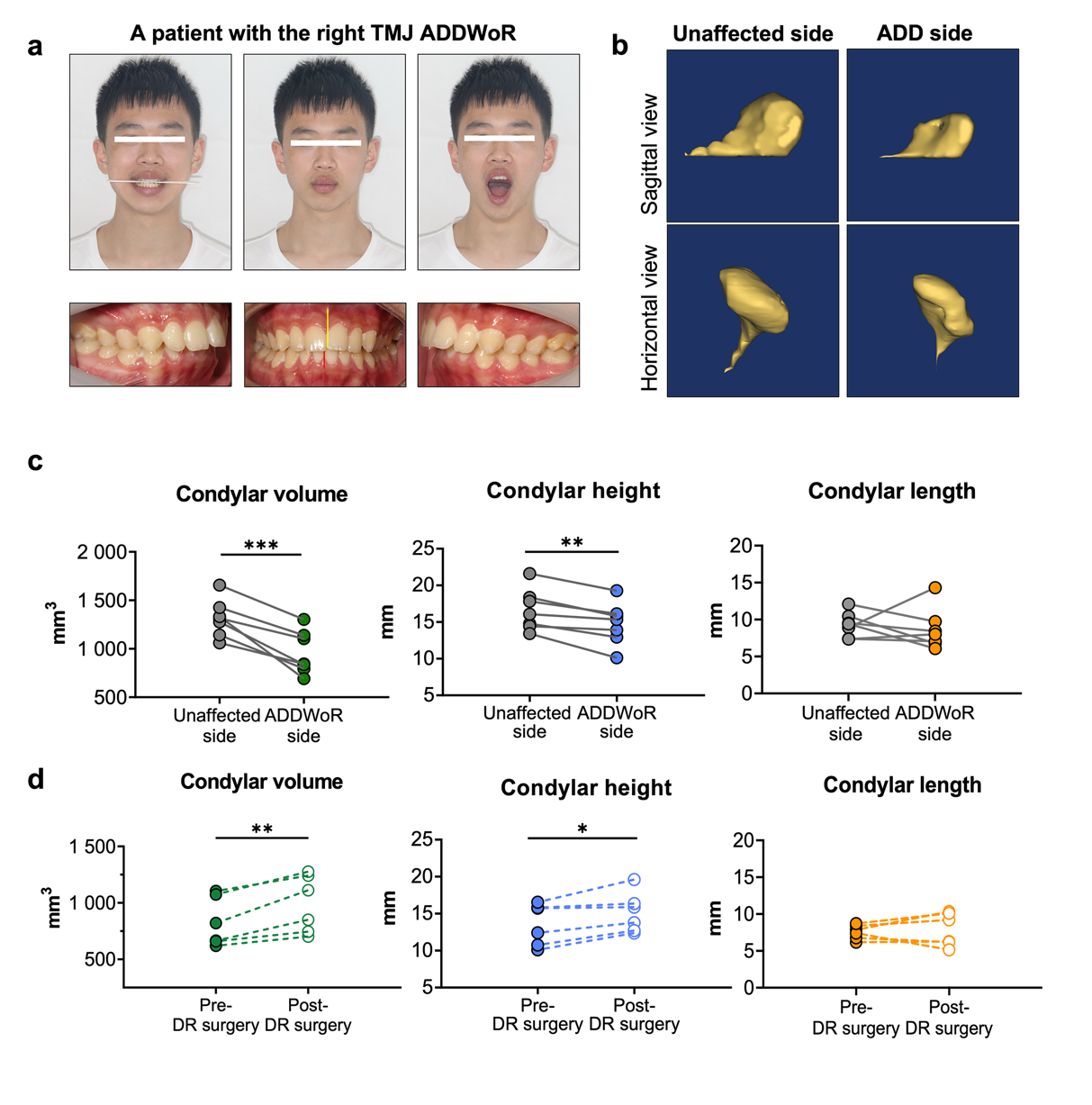
**Figure S7:** (a) Representative images of facial and intraoral photographs of an adolescent patient with the right TMJ anterior disc displacement without reduction (ADDWoR). (b) Condyles of the patients were scanned by cone beam computed tomography (CBCT) and were reconstructed for further analyses. (c) The condylar volume, condylar height, and condylar length of unilateral ADDWoR adolescent patients were analyzed. (d) 9-12 months after ADD reposition surgery, the condylar volume, condylar height, and condylar length of the surgical side were analyzed and data before and after surgery were compared. N=5. *P < 0.05, **P < 0.01, **P < 0.001.

**Figure S8:**
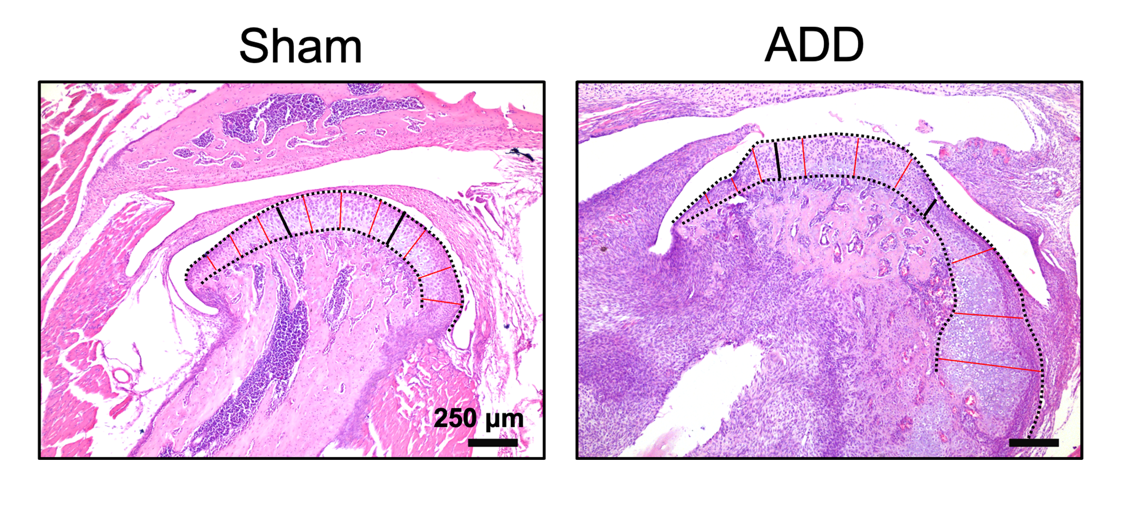
 Representative images of Cartilage thickness calculation. The average thickness of cartilage was calculated as the mean value of nine data from each TMJ sample and ultimately used for statistical analysis.
